# Supplementary material for: Insights into Autotrophic Activities and Carbon Flow in Iron-Rich Pelagic Aggregates (Iron Snow)
Source: Microorganisms. 2021 Jun 23;9(7):1368. doi: 10.3390/microorganisms9071368 (PMC8305228; doi:10.3390/microorganisms9071368)
Supplement: Supplementary file 1 [file microorganisms-09-01368-s001.zip › microorganisms-1231507-supplementary.pdf]

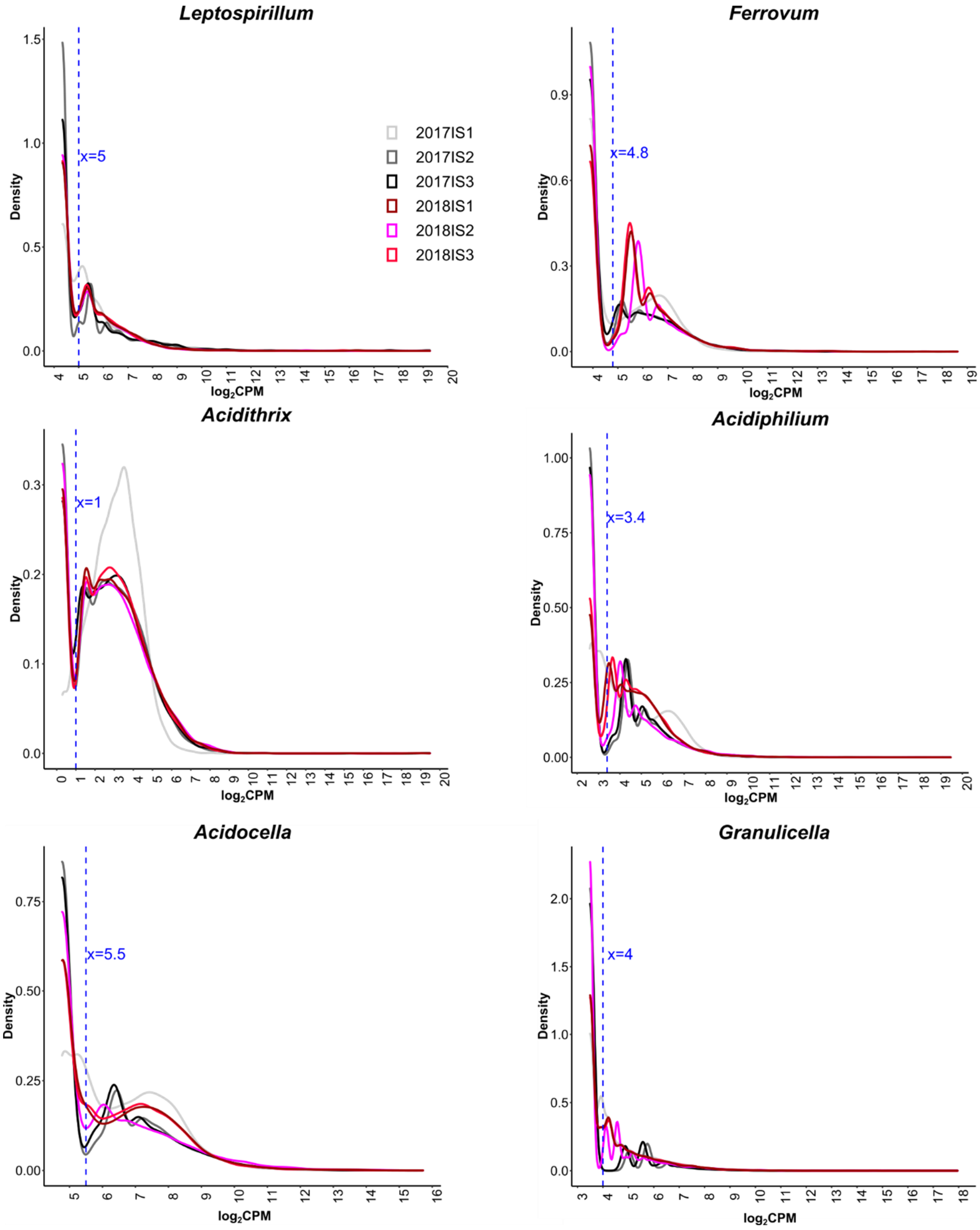

Figure S1. Density distributions of log-transformed CPM values of gene expression levels. The dashed blue line represents the cutoffs of gene expression values.

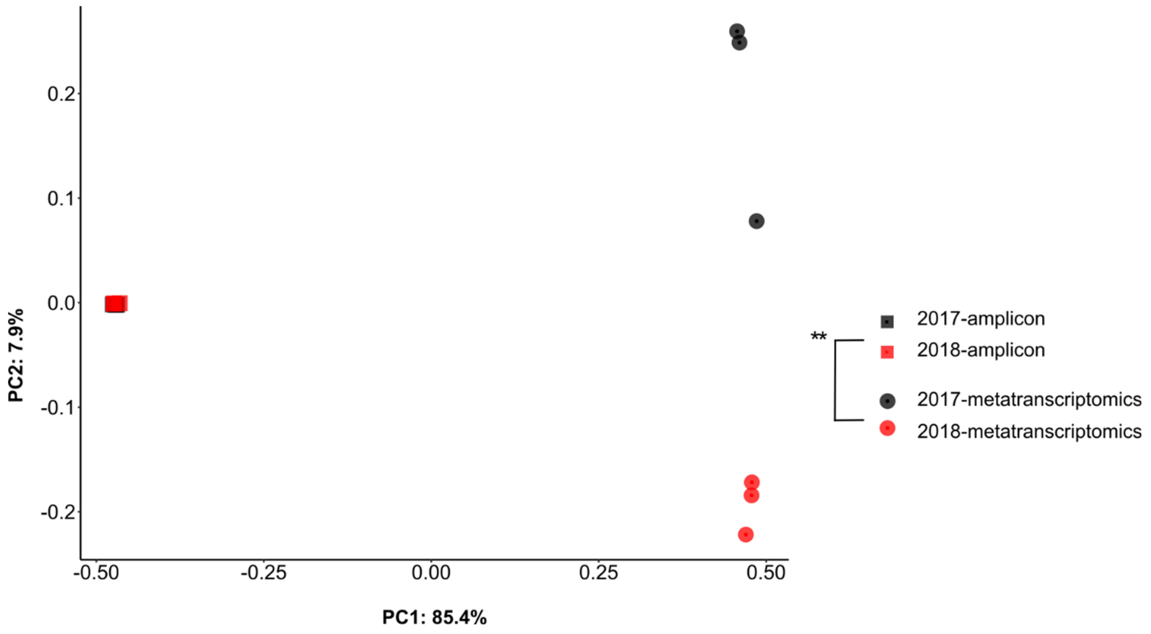

Figure S2. Principal coordinates analysis (PCoA) showing microbial community based on amplicon and mRNA sequences. Each square and circle represent an individual iron snow sample from the amplicon and mRNA sequences of metatranscriptome datasets. Black means sample from 2017 and Red means sample from 2018. Axis percentages indicate variance contribution.

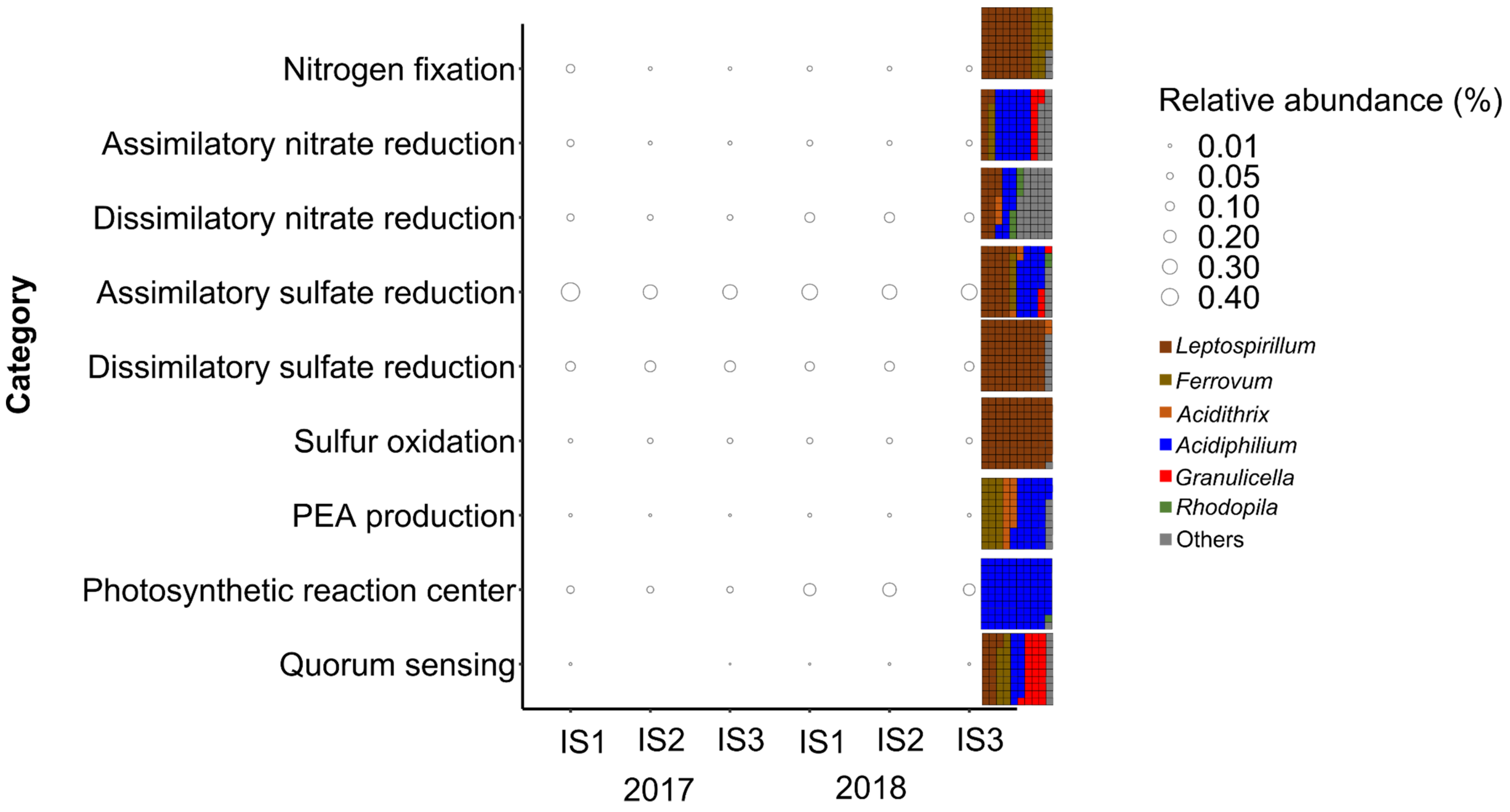

Figure S3. Functional profile of iron snow microbiome in metatranscriptome datasets. The relative abundances of functional categories in metatranscriptome datasets. Different colors represent different taxonomies linked to functional categories and only the top 6 taxonomic groups with the highest relative abundance are shown. Waffle charts represent relative abundances of assigned taxonomic groups within respective functional categories of interest.

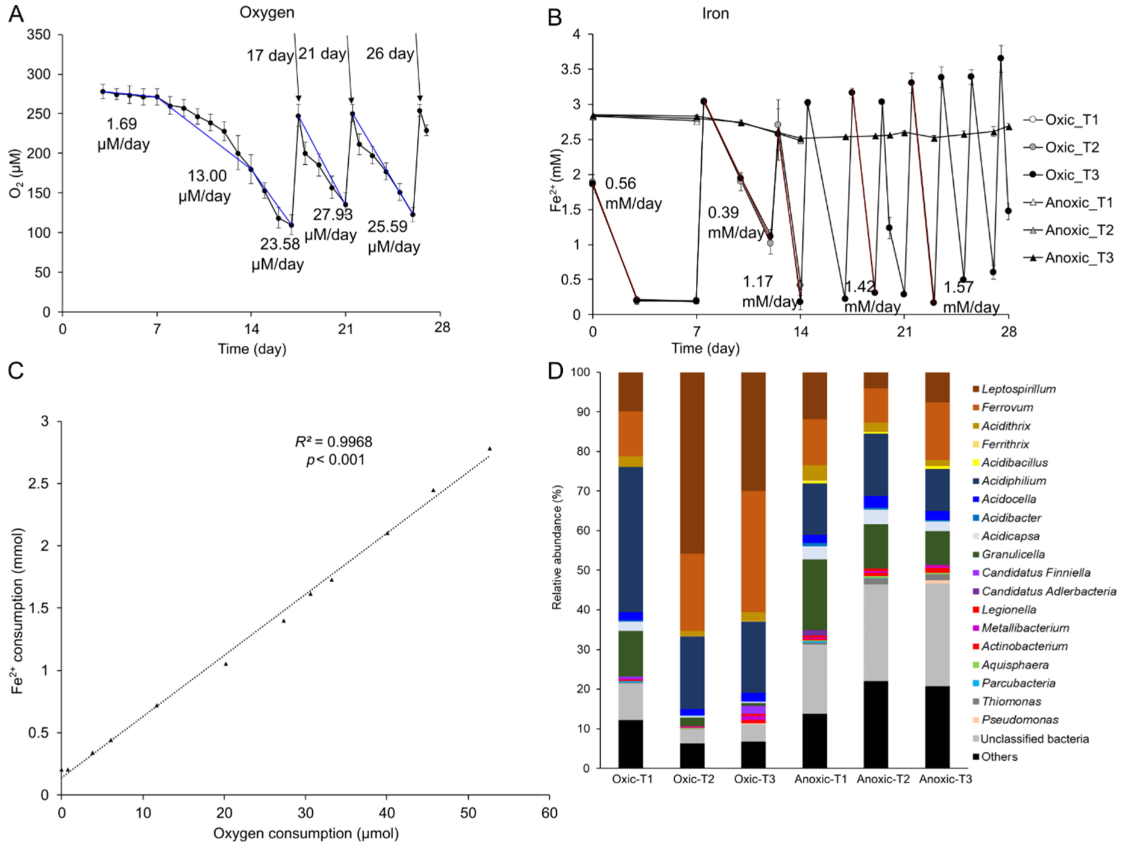

Figure S4. Oxygen, Iron concentration and microbial community composition of incubated microcosms at three timepoints (A) O_2 measured in oxic microcosms. The arrows indicate that oxygen was added to microcosms to remain sufficient oxygen for bacterial growth. (B) Fe^{2+} concentration measured in oxic and anoxic microcosms. (C) Correlation of O_2 and Fe^{2+} consumption in oxic microcosms. (D) Microbial community composition of oxic and anoxic microcosms based on 16S rRNA amplicon sequencing at three time points.

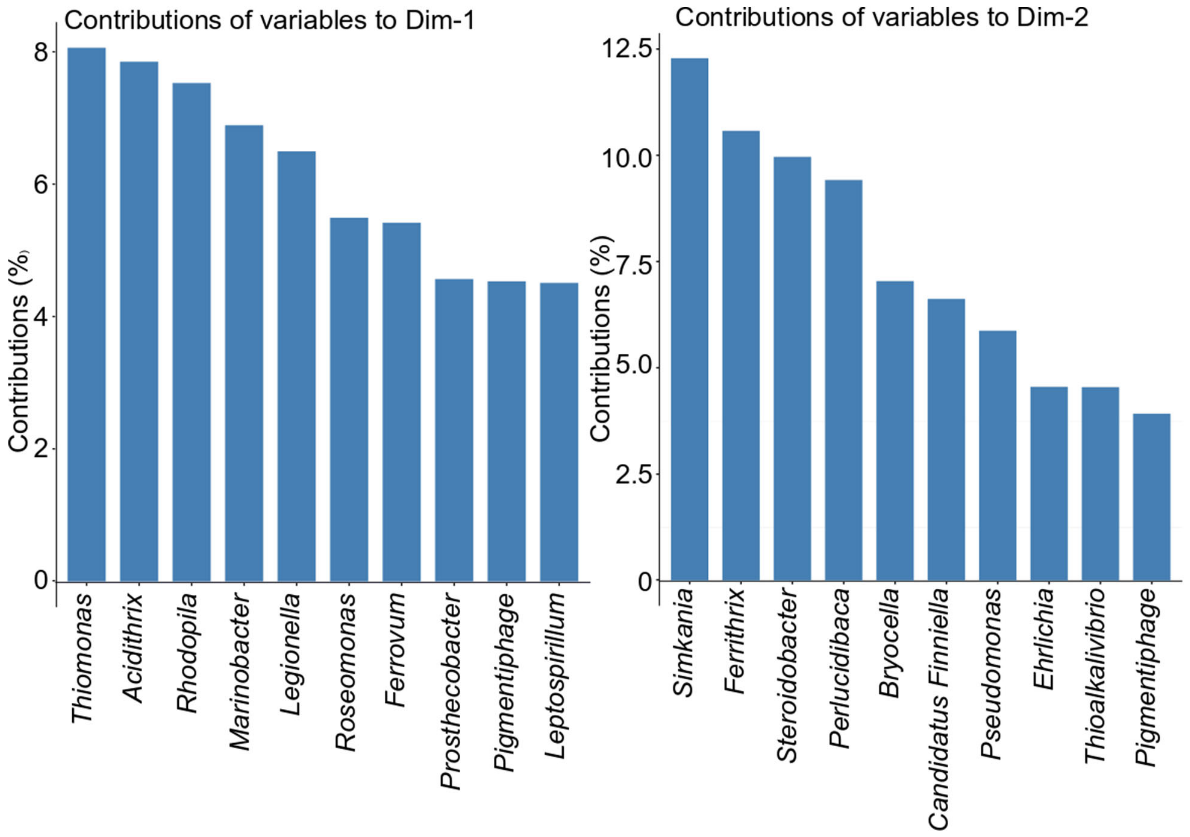

Figure S5. Taxonomic groups contributing to PCA-based ordinations of iron snow microcosms under oxic and anoxic conditions. Only top 10 contributing groups identified at genus level are shown.

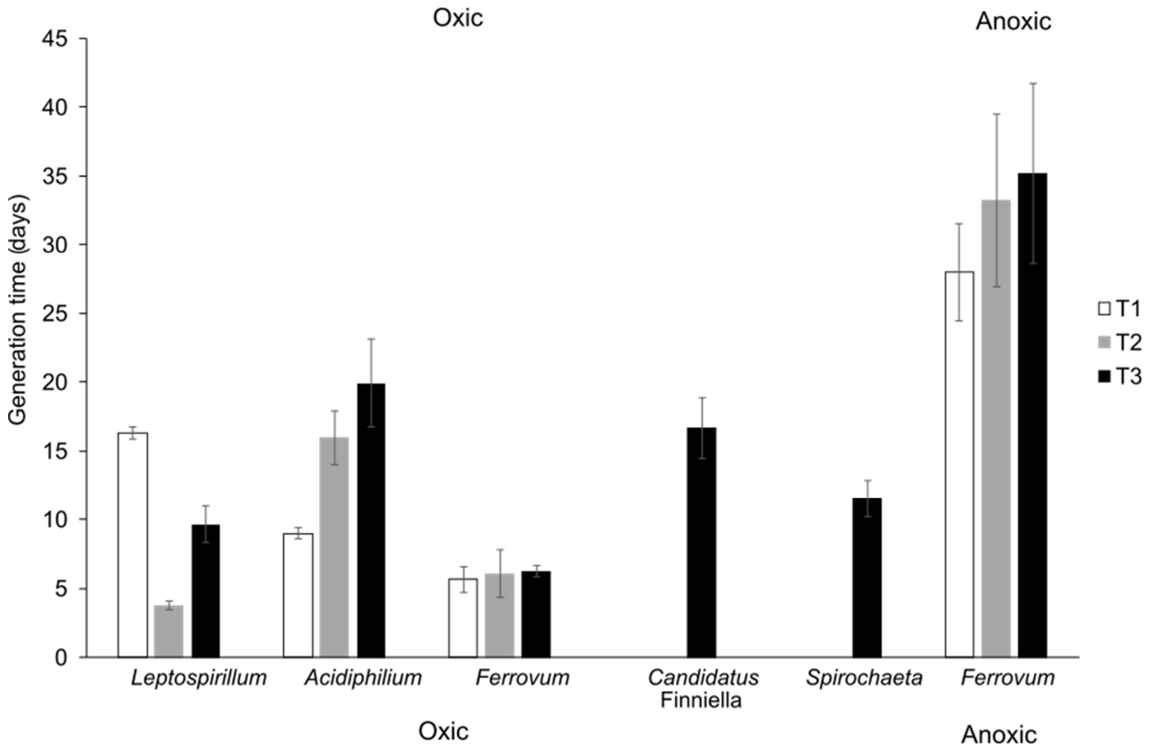

Figure S6. Growth rate of *Leptospirillum*, *Ferrovum*, *Acidiphilium*, *Spirochaeta*, *Candidatus* Finniella based on labeling intensity.

s

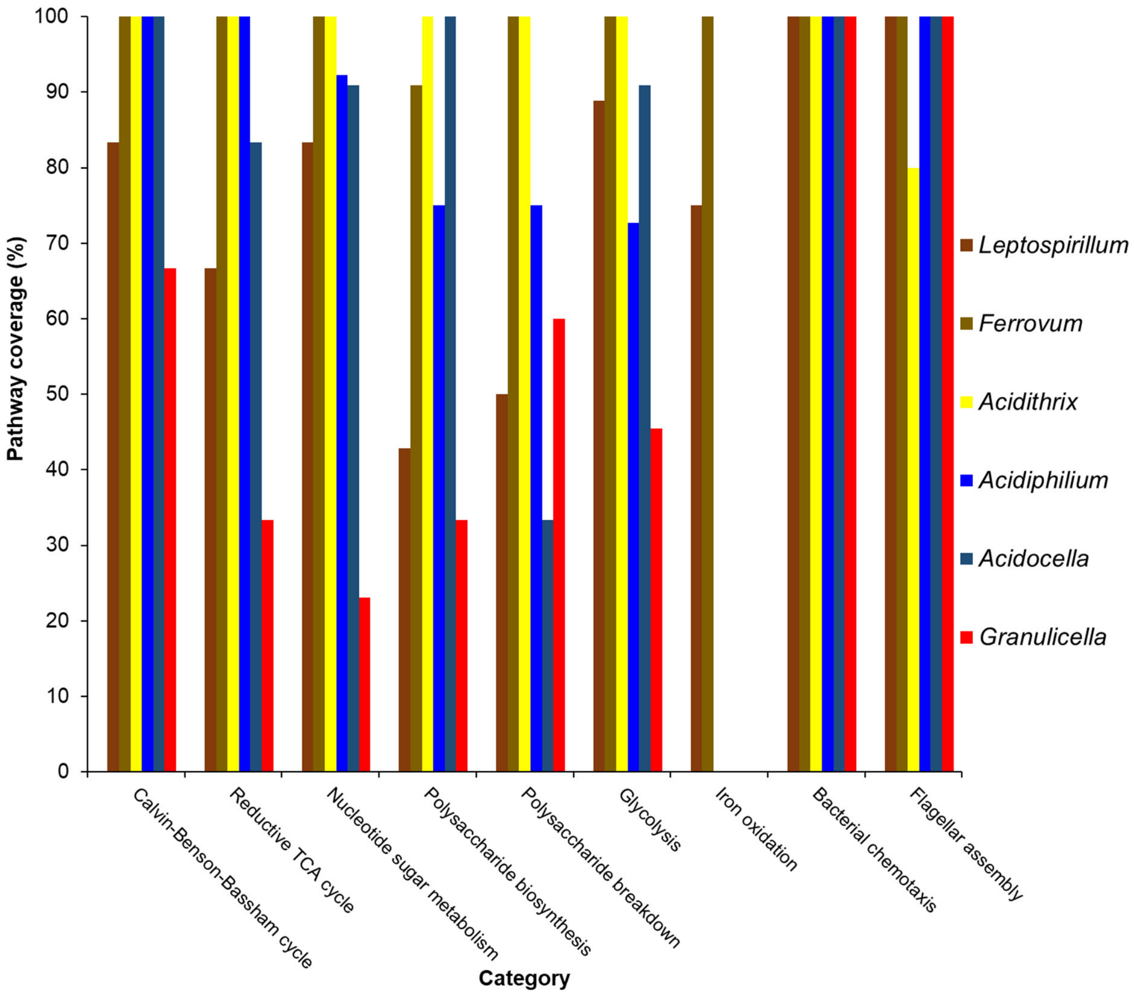

Figure S7. Pathway coverage in each genome based on mRNA sequences mapped to each genome. Different colors represent different taxonomies. The coverage ratios of pathways were calculated by dividing the numbers of expressed genes ($\log_2$CPM greater than cutoff shown in Figure S1 at least three samples) by the numbers of genes in individual bacterial genomes genes corresponding to the individual pathway.

Table S1. Reference genomes for the metabolic potential of key players.

Leptospirillum	***Ferrovum***	***Acidithrix***	***Acidiphilium***	***Acidocella***
Leptospirillum ferriphilum ML-04 **(GCA_000299235.1)**	*Ferrovum myxofaciens* strain P3G （**GCA_000735045.1)**	***Acidithrx* sp. C25**	***Acidiphilium* sp. C61** **(GCA_902712915.1**)	***Acidocella* sp. C78**
Leptospirillum ferrooxidans C2-3 **(GCA_000284315.1)**	*Ferrovum* sp. 21-44-67 **(GCA_002255765.1)**	*Acidithrix ferrooxidans* strain Py-F3 **(GCA_000949295.1)**	*Acidiphilium cryptum* JF-5 **(GCA_000016725.1)**	*Acidocella facilis* ATCC 35904 **(GCA_000687875.1)**
Leptospirillum rubarum **(GCA_000205145.2)**	*Ferrovum* sp. 34-44-207 **(GCA_002279055.1)**		*Acidiphilium multivorum* AIU301 （**GCA_000202835.1)**	*Acidocella aminolytica* 101 = DSM 11237 **(GCA_900129125.1)**
Leptospirillum sp. Group II 'C75' **(GCA_000262365.1)**	*Ferrovum* sp. 37-45-19 **(GCA_002279305.1)**		*Acidiphilium angustum* ATCC35903 **(GCA_000701585.1)**	*Acidocella* sp. 20-57-95 **(GCA_002255575.1)**
Leptospirillum sp. Group II 'CF-1' **(GCA_001186405.1)**	*Ferrovum* sp. JA12 **(GCA_001431705.1)**		*Acidiphilium rubrum* ATCC 35905 **(GCA_900156265.1)**	*Acidocella* sp. 20-58-15 **(GCA_002255785.1)**
Leptospirillum sp. Group IV **(GCA_000496115.1)**	*Ferrovum* sp. PN-J185 **(GCA_001581925.1)**		*Acidiphilium* sp. JA12-A1 **(GCA_000724705.2)**	*Acidocella* sp. 20-61-6 **(GCA_002255625.1)**
	Ferrovum sp. Z-31 **(GCA_001581915.1)**		*Acidiphilium* sp. PM **(GCA_000219295.2)**	*Acidocella* sp. 20-63-7 **(GCA_002255455.1)**
				Acidocella sp. 21-58-7 **(GCA_002255715.1)**
				Acidocella sp. 35-58-6 **(GCA_002281005.1)**
				Acidocella sp. MX-AZ02 **(GCA_000306035.1)**

Granulicella	
*Granulicella_tundricola*_MP5ACTX9 (**GCA_000178975.2)**	*Granulicella_sibirica* **(GCA_004115155.1)**
*Granulicella_rosea*_strain_DSM_18704 (**GCA_900188085.1**)	*Granulicella_pectinivorans*_DSM21001 **(GCA_900114625.1)**
Granulicella sp.UBA5172 **(GCA_002413385.1)**	*Granulicella_mallensis*_MP5ACTX8 **(GCA_000178955.2)**
Granulicella sp.UBA4739 **(GCA_002403945.1)**	*Granulicella* sp. isolate bog_445 **(GCA_003162875.1)**
Granulicella sp.SCN_62-9 **(GCA_001725305.1)**	*Granulicella* sp. isolate bog_405 **(GCA_003142215.1)**
*Granulicella sp.*S190 **(GCA_009766095.1)**	*Granulicella* sp. isolate bog_396 **(GCA_003159875.1)**
Granulicella sp.S156 **(GCA_009766105.1)**	*Granulicella* sp.L46 (**GCA_009765785.1**)
Granulicella sp.L60 **(GCA_009765825.1)**	*Granulicella* sp. isolate bog_395 **(GCA_003159515.1)**
Granulicella sp.L56 **(GCA_009765835.1)**	*Granulicella* sp. isolate bog_115 **(GCA_003168875.1)**
Granulicella sp.isolate palsa_288 **(GCA_003133205.1)**	*Granulicella* sp. isolate bog_394 **(GCA_003159575.1)**
Granulicella sp.isolate palsa_287 **(GCA_003132145.1)**	*Granulicella* sp.GAS466 (GCA_003751275.1)
Granulicella sp.isolate bog_404 **(GCA_003142235.1)**	
Granulicella sp.isolate bog_402 **(GCA_003159275.1)**	
Granulicella sp.isolate bog_392 **(GCA_003159855.1)**	
Granulicella sp.isolate bog_369 **(GCA_003165175.1)**	
Granulicella sp.isolate bog_326 **(GCA_003155975.1)**	
Granulicella sp.isolate bog_259 **(GCA_003134355.1)**	
Granulicella sp.isolate bog_230 **(GCA_003138115.1)**	
Granulicella sp.isolate bog_215 **(GCA_003138715.1)**	
Granulicella sp.isolate bog_170 **(GCA_003165995.1)**	
Granulicella sp.isolate bog_168 **(GCA_003166015.1)**	
Granulicella sp.isolate bog_149 **(GCA_003166755.1)**	
Granulicella sp.isolate bog_99 **(GCA_003161035.1)**	
Granulicella sp.isolate bog_97 **(GCA_003161365.1)**	
Granulicella sp.isolate bog_95 **(GCA_003161075.1)**	
Granulicella sp.isolate bog_213 **(GCA_003138735.1)**	

Table S2. Concentrations of sulfate (mM) in 2017 and 2018.

**Depth (m)**	1	1.5	2	2.5	3	3.5	4	4.5	5	5.5	6	6.5
2017	18.60	19.10	19.64	17.48	19.53	18.14	17.37	18.02	19.14	17.37	17.02	16.17
2018	18.25	17.68	17.68	17.33	17.16	19.34	16.82	16.07	16.01	15.84	15.90	16.87

Table S3. Relative abundance of iron cycling bacteria based on 16S rRNA amplicon datasets, mRNA/ rRNA sequences from metatranscriptome datasets.

	**Amplicon**		**Metatranscriptome-mRNA**		**Metatranscriptome-rRNA**	
Genus	2017	2018	2017	2018	2017	2018
Leptospirillum	20.28	5.11	51.38	14.91	73.77	38.57
Ferrovum	13.13	15.35	7.14	6.35	7.72	13.62
Acidithrix	7.60	5.82	8.71	11.81	0	0
Acidithiobacillus	0.00	0.00	0.42	0.28	0	0
Ferrimicrobium	0.00	0.00	0.23	0.26	0	0
Ferrithrix	1.55	0.57	0.24	0.21	0.05	0.12
Sideroxydans	0	0	0.10	0.10	0	0
Acidibacillus	0.05	0.36	0. 02	0.06	0	0
Acidiphilium	21.49	30.20	14.53	27.51	8.86	23.37
Acidocella	0.72	0.32	0.65	0.78	0.20	0.56
Acidibacter	0.50	0.26	0	0	0.44	0.57
Acidicapsa	1.45	6.52	0		0.27	2.59
Acidipila	0	0	0.34	1.32	0	0
Acidobacterium	0	0	0.15	0.50	0	0

Table S4. Relative abundance of eukaryotes at class level based on 18S rRNA sequences from metatranscriptome datasets.

Class	2017IS1	2017IS2	2017IS3	2018IS1	2018IS2	2018IS3
Stramenopiles	74.59	79.57	76.48	88.52	90.96	91.73
Holozoa	13.96	10.45	10.85	2.13	1.44	1.52
Discoba	4.66	3.08	4.45	0.78	2.44	1.90
Chloroplastida	2.07	1.98	3.38	3.59	2.77	2.50
Alveolata	1.88	1.77	1.77	2.30	0.88	0.94
Nucletmycea	1.52	1.87	1.99	1.41	1.15	0.73
Others	1.32	1.28	1.08	1.27	0.36	0.68

Table S5. Sequence processing statistics of metatranscriptome datasets.

Sample	No. of raw read pairs	No. of trimmed read pairs	No. of QCed read pairs	No. of assembled sequences by PEAR	No. of mRNA derived sequences	No. of bacterial mRNA taxonomically assigned sequences	No. of bacterial mRNA KEGG annotated sequences	No. of archeal mRNA sequences taxonomically assigned	No. of archeal KEGG annotated sequences	No. of eukaryotic mRNA sequences taxonomically assigned	No. of eukaryotic KEGG annotated sequences
2017IS_1	51816264	51195218	50207959	46164876	6035123	1140375	657565	5881	1898	9808	5829
2017IS_2	73592955	72797930	71901366	68679466	3944159	415400	248908	986	336	8170	5210
2017IS_3	103305514	102613435	101251058	95644871	4186572	502000	296237	1096	344	12487	8067
2018IS_1	110075121	109367353	108270534	101784354	6445524	445000	300189	1838	505	41335	28539
2018IS_2	102491388	102191604	101135842	94596914	4269577	302500	209959	1399	401	32093	21744
2018IS_3	101489570	100038832	100038832	95178726	5728224	414000	278626	1744	518	31318	21646

Taxonomic assignments of mRNA sequences using diamond and NCBI RefSeq as reference database, diamond output was parsed using megan and applying default settings for the implemented lowest common ancestor algorithm.

Functional annotations using KEGG database by parsing diamond output via self-made python script using available mapping files.

Table S6. List of KOs for functional categories of interest based on assignments to KEGG.

Category	KO	Function
Iron oxidation	cyt572	
Iron oxidation	cyt579	
Iron oxidation	cyc2	
Iron reduction	K17247	methionine sulfoxide reductase heme-binding subunit
Iron reduction	K11811	arsenical resistance protein ArsH
CBB cycle	K01601	ribulose-bisphosphate carboxylase large chain
CBB cycle	K01602	ribulose-bisphosphate carboxylase small chain
rTCA cycle	K00244	fumarate reductase flavoprotein subunit
rTCA cycle	K00245	fumarate reductase iron-sulfur subunit
rTCA cycle	K00246	fumarate reductase subunit C
rTCA cycle	K00247	fumarate reductase subunit D
rTCA cycle	K01648	ATP citrate (pro-S)-lyase
rTCA cycle	K15230	ATP-citrate lyase alpha-subunit
rTCA cycle	K15231	ATP-citrate lyase beta-subunit
rTCA cycle	K15232	ATP-citrate lyase beta-subunit
rTCA cycle	K15233	citryl-CoA synthetase small subunit
rTCA cycle	K15234	citryl-CoA lyase
rTCA cycle	K18556	NADH-dependent fumarate reductase subunit A
rTCA cycle	K18557	NADH-dependent fumarate reductase subunit B
rTCA cycle	K18558	NADH-dependent fumarate reductase subunit C
rTCA cycle	K18559	NADH-dependent fumarate reductase subunit D
rTCA cycle	K18560	NADH-dependent fumarate reductase subunit E
rTCA cycle	K18859	succinate dehydrogenase / fumarate reductase, subunit D
rTCA cycle	K18860	succinate dehydrogenase / fumarate reductase, subunit D
rTCA cycle	K15230	ATP-citrate lyase alpha-subunit
rTCA cycle	K00174	2-oxoglutarate/2-oxoacid ferredoxin oxidoreductase subunit
rTCA cycle	K00175	2-oxoglutarate/2-oxoacid ferredoxin oxidoreductase subunit
rTCA cycle	K00176	2-oxoglutarate ferredoxin oxidoreductase subunit delta
rTCA cycle	K00177	2-oxoglutarate ferredoxin oxidoreductase subunit gamma
Nucleotide sugar	K00012	UDPglucose 6-dehydrogenase
Nucleotide sugar	K00849	galactokinase
Nucleotide sugar	K00963	UTP--glucose-1-phosphate uridylyltransferase
Nucleotide sugar	K00965	UDPglucose--hexose-1-phosphate uridylyltransferase
Nucleotide sugar	K01784	UDP-glucose 4-epimerase
Nucleotide sugar	K01835	phosphoglucomutase
Nucleotide sugar	K01854	UDP-galactopyranose mutase
Nucleotide sugar	K12447	UDP-sugar pyrophosphorylase
Nucleotide sugar	K15779	phosphoglucomutase / phosphopentomutase
Nucleotide sugar	K16190	glucuronokinase
Nucleotide sugar	K17716	UDP-glucose 4-epimerase

Nucleotide sugar	K18677	galacturonokinase
Exopolysaccharide biosynthesis	K00640	serine O-acetyltransferase
Exopolysaccharide biosynthesis	K00694	cellulose synthase (UDP-forming)
Exopolysaccharide biosynthesis	K01991	polysaccharide biosynthesis/export protein
Exopolysaccharide biosynthesis	K03606	putative colanic acid biosysnthesis
Exopolysaccharide biosynthesis	K05851	adenylate cyclase, class 1
Exopolysaccharide biosynthesis	K11931	poly-beta-1,6-N-acetyl-D-glucosamine N-deacetylase
Exopolysaccharide biosynthesis	K11935	biofilm PGA synthesis protein PgaA
Exopolysaccharide biosynthesis	K11936	icaA; poly-beta-1,6-N-acetyl-D-glucosamine synthase
Exopolysaccharide biosynthesis	K11937	biofilm PGA synthesis protein PgaD
Exopolysaccharide biosynthesis	K12992	rhamnosyltransferase
Exopolysaccharide biosynthesis	K16554	polysaccharide biosynthesis transport protein
Exopolysaccharide biosynthesis	K16011	mannose-1-phosphate guanylyltransferase / mannose-6-phosphate isomerase [EC:2.7.7.13 5.3.1.8]
Exopolysaccharide biosynthesis	K20987	polysaccharide biosynthesis/export protein PslD
Exopolysaccharide biosynthesis	K20997	polysaccharide biosynthesis protein PslA
Exopolysaccharide biosynthesis	K20998	polysaccharide biosynthesis protein PslE
Exopolysaccharide biosynthesis	K20999	polysaccharide biosynthesis protein PslF
Exopolysaccharide biosynthesis	K21000	polysaccharide biosynthesis protein PslG
Exopolysaccharide biosynthesis	K21001	polysaccharide biosynthesis protein PslH
Exopolysaccharide biosynthesis	K21002	polysaccharide biosynthesis protein PslI
Exopolysaccharide biosynthesis	K21003	polysaccharide biosynthesis protein PslJ
Exopolysaccharide biosynthesis	K21004	polysaccharide biosynthesis protein PslK
Exopolysaccharide biosynthesis	K21005	polysaccharide biosynthesis protein PslL
Exopolysaccharide biosynthesis	K21006	polysaccharide biosynthesis protein PelA
Exopolysaccharide biosynthesis	K21007	polysaccharide biosynthesis protein PelB
Exopolysaccharide biosynthesis	K21008	polysaccharide biosynthesis protein PelC
Exopolysaccharide biosynthesis	K21009	polysaccharide biosynthesis protein PelD
Exopolysaccharide biosynthesis	K21010	polysaccharide biosynthesis protein PelE
Exopolysaccharide biosynthesis	K21011	polysaccharide biosynthesis protein PelF
Exopolysaccharide biosynthesis	K21012	polysaccharide biosynthesis protein PelG
Glycolysis	K00844	hexokinase
Glycolysis	K00845	glucokinase
Glycolysis	K00886	polyphosphate glucokinase
Glycolysis	K00850	6-phosphofructokinase 1
Glycolysis	K16370	6-phosphofructokinase 2
Glycolysis	K21071	ATP-dependent phosphofructokinase / diphosphate-dependent phosphofructokinase
Glycolysis	K24182	6-phosphofructokinase
Glycolysis	K00873	pyruvate kinase
Glycolysis	K12406	pyruvate kinase isozymes R/L
Glycolysis	K00616	transaldolase
Glycolysis	K13810	transaldolase / glucose-6-phosphate isomerase

Exopolysaccharide breakdown	K00731	glycoprotein-N-acetylgalactosamine 3-beta-galactosyltransferase
Exopolysaccharide breakdown	K01176	alpha-amylase
Exopolysaccharide breakdown	K01177	beta-amylase
Exopolysaccharide breakdown	K01178	glucoamylase
Exopolysaccharide breakdown	K01179	endoglucanase
Exopolysaccharide breakdown	K01181	endo-1,4-beta-xylanase
Exopolysaccharide breakdown	K01187	alpha-glucosidase
Exopolysaccharide breakdown	K01188	beta-glucosidase
Exopolysaccharide breakdown	K01183	chitinase
Exopolysaccharide breakdown	K01191	alpha-mannosidase
Exopolysaccharide breakdown	K01195	beta-glucuronidase
Exopolysaccharide breakdown	K01728	pectate lyase
Exopolysaccharide breakdown	K01729	poly(beta-D-mannuronate) lyase
Exopolysaccharide breakdown	K05343	maltose alpha-D-glucosyltransferase / alpha-amylase
Exopolysaccharide breakdown	K05349	beta-glucosidase
Exopolysaccharide breakdown	K05350	beta-glucosidase
Exopolysaccharide breakdown	K06119	sulfoquinovosyltransferase
Exopolysaccharide breakdown	K07405	alpha-amylase
Exopolysaccharide breakdown	K12047	maltase-glucoamylase
Exopolysaccharide breakdown	K13881	bestrophin
Exopolysaccharide breakdown	K19357	cellulase
Exopolysaccharide breakdown	K19551	pectate lyase C
Exopolysaccharide breakdown	K20542	endoglucanase
Exopolysaccharide breakdown	K20547	basic endochitinase B
Exopolysaccharide breakdown	K21366	beta-1,3-galactosyltransferase
Exopolysaccharide breakdown	K21574	glucan 1,4-alpha-glucosidase
Exopolysaccharide breakdown	K22092	beta-glucosidase
Exopolysaccharide breakdown	K22539	pectate lyase
Exopolysaccharide breakdown	K22451	4-alpha-glucanotransferase
Flagellar biosynthesis	K02386	flagellar basal body P-ring formation protein FlgA
Flagellar biosynthesis	K02387	flagellar basal-body rod protein FlgB
Flagellar biosynthesis	K02388	flagellar basal-body rod protein FlgC
Flagellar biosynthesis	K02389	flagellar basal-body rod modification protein FlgD
Flagellar biosynthesis	K02390	flagellar hook protein FlgE
Flagellar biosynthesis	K02391	flagellar basal-body rod protein FlgF
Flagellar biosynthesis	K02392	flagellar basal-body rod protein FlgG
Flagellar biosynthesis	K02393	flagellar L-ring protein FlgH
Flagellar biosynthesis	K02394	flagellar P-ring protein FlgI
Flagellar biosynthesis	K02396	flagellar hook-associated protein 1
Flagellar biosynthesis	K02397	flagellar hook-associated protein 3 FlgL
Flagellar biosynthesis	K02400	flagellar biosynthesis protein FlhA
Flagellar biosynthesis	K02401	flagellar biosynthesis protein FlhB
Flagellar biosynthesis	K02402	flagellar transcriptional activator FlhC

Flagellar biosynthesis	K02403	flagellar transcriptional activator FlhD
Flagellar biosynthesis	K02405	RNA polymerase sigma factor FliA
Flagellar biosynthesis	K02406	flagellin
Flagellar biosynthesis	K02407	flagellar hook-associated protein 2
Flagellar biosynthesis	K02408	flagellar hook-basal body complex protein FliE
Flagellar biosynthesis	K02409	flagellar M-ring protein FliF
Flagellar biosynthesis	K02410	flagellar motor switch protein FliG
Flagellar biosynthesis	K02411	flagellar assembly protein FliH
Flagellar biosynthesis	K02412	flagellum-specific ATP synthase
Flagellar biosynthesis	K02414	flagellar hook-length control protein FliK
Flagellar biosynthesis	K02416	flagellar motor switch protein FliM
Flagellar biosynthesis	K02417	flagellar motor switch protein FliN
Flagellar biosynthesis	K02418	flagellar protein FliO/FliZ
Flagellar biosynthesis	K02419	flagellar biosynthesis protein FliP
Flagellar biosynthesis	K02420	flagellar biosynthesis protein FliQ
Flagellar biosynthesis	K02421	flagellar biosynthesis protein FliR
Flagellar biosynthesis	K02422	flagellar secretion chaperone FliS
Flagellar biosynthesis	K02556	chemotaxis protein MotA
Flagellar biosynthesis	K02557	chemotaxis protein MotB
Bacterial chemotaxis	K00575	chemotaxis protein methyltransferase CheR
Bacterial chemotaxis	K02410	flagellar motor switch protein FliG
Bacterial chemotaxis	K02416	flagellar motor switch protein FliM
Bacterial chemotaxis	K02417	flagellar motor switch protein FliN
Bacterial chemotaxis	K03406	methyl-accepting chemotaxis protein
Bacterial chemotaxis	K03407	chemotaxis family, sensor kinase CheA
Bacterial chemotaxis	K03408	purine-binding chemotaxis protein CheW
Bacterial chemotaxis	K03410	chemotaxis protein CheC
Bacterial chemotaxis	K03411	chemotaxis protein CheD
Bacterial chemotaxis	K03412	chemotaxis family, protein-glutamate methylesterase/glutaminase
Bacterial chemotaxis	K03413	chemotaxis family, chemotaxis protein CheY
Bacterial chemotaxis	K03414	chemotaxis protein CheZ
Bacterial chemotaxis	K03415	chemotaxis family, chemotaxis protein CheV
Bacterial chemotaxis	K03776	aerotaxis receptor
Bacterial chemotaxis	K05874	methyl-accepting chemotaxis protein I
Bacterial chemotaxis	K05875	methyl-accepting chemotaxis protein II
Bacterial chemotaxis	K05876	methyl-accepting chemotaxis protein III
Bacterial chemotaxis	K05877	methyl-accepting chemotaxis protein IV
Bacterial chemotaxis	K10439	ribose transport system substrate-binding protein
Bacterial chemotaxis	K12368	dipeptide transport system substrate-binding protein
Bacterial chemotaxis	K13924	chemotaxis family, CheB/CheR fusion protein
Nitrogen fixation	K02586	nitrogenase molybdenum-iron protein alpha chain
Nitrogen fixation	K02591	nitrogenase molybdenum-iron protein beta chain
Nitrogen fixation	K02588	nitrogenase iron protein NifH

Assimilatory nitrate reduction	K00366	ferredoxin-nitrite reductase
Assimilatory nitrate reduction	K00367	ferredoxin-nitrate reductase
Assimilatory nitrate reduction	K00360	assimilatory nitrate reductase electron transfer subunit
Assimilatory nitrate reduction	K17877	nitrite reductase (NAD(P)H)
Assimilatory nitrate reduction	K00372	assimilatory nitrate reductase catalytic subunit
Assimilatory nitrate reduction	K10534	nitrate reductase (NAD(P)H)
Dissimilatory nitrate reduction	K00362	nitrite reductase (NADH) large subunit
Dissimilatory nitrate reduction	K00363	nitrite reductase (NADH) small subunit
Dissimilatory nitrate reduction	K00370	nitrate reductase / nitrite oxidoreductase, alpha subunit
Dissimilatory nitrate reduction	K00371	nitrate reductase / nitrite oxidoreductase, beta subunit
Dissimilatory nitrate reduction	K00374	nitrate reductase gamma subunit
Dissimilatory nitrate reduction	K02567	nitrate reductase (cytochrome)
Dissimilatory nitrate reduction	K02568	nitrate reductase (cytochrome), electron transfer subunit
Sulfur oxidation	K17222	L-cysteine S-thiosulfotransferase
Sulfur oxidation	K11180	dissimilatory sulfite reductase alpha subunit
Sulfur oxidation	K11181	dissimilatory sulfite reductase beta subunit
Assimilatory sulfate reduction	K00380	sulfite reductase (NADPH) flavoprotein alpha-component
Assimilatory sulfate reduction	K00381	sulfite reductase (NADPH) hemoprotein beta-component
Assimilatory sulfate reduction	K00390	phosphoadenosine phosphosulfate reductase
Assimilatory sulfate reduction	K00392	sulfite reductase (ferredoxin)
Assimilatory sulfate reduction	K00860	adenylylsulfate kinase
Assimilatory sulfate reduction	K00955	bifunctional enzyme CysN/CysC
Assimilatory sulfate reduction	K00956	sulfate adenylyltransferase subunit 1
Assimilatory sulfate reduction	K00957	sulfate adenylyltransferase subunit 2
Assimilatory sulfate reduction	K00958	sulfate adenylyltransferase
Assimilatory sulfate reduction	K13811	3'-phosphoadenosine 5'-phosphosulfate synthase
Dissimilatory sulfate reduction	K00394	adenylylsulfate reductase, subunit A
Dissimilatory sulfate reduction	K00395	adenylylsulfate reductase, subunit B
Dissimilatory sulfate reduction	K00958	sulfate adenylyltransferase
Dissimilatory sulfate reduction	K11180	dissimilatory sulfite reductase alpha subunit
Dissimilatory sulfate reduction	K11181	dissimilatory sulfite reductase beta subunit
PEA production	K01593	aromatic-L-amino-acid/L-tryptophan decarboxylase
PEA production	K22427	phenylalanine decarboxylase
Photosynthetic reaction center	K08929	photosynthetic reaction center M subunit
Photosynthetic reaction center	K08928	photosynthetic reaction center L subunit
Photosynthetic reaction center	K08927	light-harvesting complex 1 beta chain
Photosynthetic reaction center	K08926	light-harvesting complex 1 alpha chain
Photosynthetic reaction center	K13991	photosynthetic reaction center H subunit
Photosynthetic reaction center	K13992	photosynthetic reaction center cytochrome c subunit
Photosynthetic reaction center	K13994	photosynthetic reaction center PufX protein

Table S7. Numbers of expressed genes in individual genomes of key players.

Genomes	Number of expressed genes	mRNA mapping ratio (%) of expressed genes to taxonomical mRNA sequences at genus level
Leptospirillum ferrooxidans C2-3	1102	*Leptospirillum*
Leptospirillum rubarum	54	
Leptospirillum sp. Group II 'CF-1'	64	
Leptospirillum ferriphilum ML-04	29	0.9%-1.2%
Leptospirillum sp. Group II 'C75'	21	
Leptospirillum sp. Group IV	140	
Ferrovum sp. Z-31	2002	*Ferrovum*
Ferrovum myxofaciens strain P3G	1826	
Ferrovum sp. JA12	273	1.3-1.5%
Ferrovum sp. 37-45-19	165	
Ferrovum sp. 21-44-67	145	
Ferrovum sp. 34-44-207	145	
Ferrovum sp. PN-J185	10	
Acidithrix ferroxidans Py-F3	2721	*Acidithrix*
***Acidithrix* sp. C25**	3008	2.8%-4.1%
Acidiphilium rubrum ATCC 35905	2776	*Acidiphilium*
Acidiphilium angustum ATCC 35903	2692	
***Acidiphilium* sp. C61**	2991	
Acidiphilium sp. JA12-A1	691	
Acidiphilium sp. PM	633	4.6%-7.3%
Acidiphilium cryptum JF-5	722	
Acidiphilium multivorum AIU301	702	
***Acidocella* sp. C78**	2936	*Acidocella*
Acidocella sp. 21-58-7	95	
Acidocella facilis ATCC 35904	108	
Acidocella sp. 20-61-6	130	
Acidocella sp. 20-58-15	32	1.7%-2.0%
Acidocella aminolytica DSM 11237	83	
Acidocella sp. MX-AZ02	77	
Acidocella sp. 20-57-95	69	
Acidocella sp. 20-63-7	42	
Acidocella sp. 35-58-6	38	
Granulicella sp. L56	86	*Granulicella*
Granulicella sp. L46	542	
Granulicella mallensis MP5ACTX8	151	4.2%-5.0%
Granulicella sp. UBA5172	643	
Granulicella sp. UBA4739	452	

Granulicella sp. isolate palsa_287	145
Granulicella sp. isolate bog_99	48
Granulicella sp. isolate bog_396	59
Granulicella sp. isolate bog_392	423
Granulicella sp. isolate bog_97	398
Granulicella sp. isolate bog_402	397
Granulicella sp. isolate bog_215	370
Granulicella sp. isolate bog_210	434
Granulicella sp. isolate bog_326	34
Granulicella sp. isolate bog_395	63
Granulicella sp. isolate palsa_288	145
Granulicella sp. L60	125
Granulicella rosea strain DSM 18704	144
Granulicella sp. S156	148
Granulicella pectinivorans DSM 21001	129
Granulicella sp. isolate bog_404	57
Granulicella sp. S190	81
Granulicella tundricola MP5ACTX9	121
Granulicella sp. GAS466	121
Granulicella sp. isolate bog_394	54
Granulicella sp. isolate bog_170	29
Granulicella sibirica	96
Granulicella sp. isolate bog_168	30
Granulicella sp. isolate bog_259	30
Granulicella sp. isolate bog_213	46
Granulicella sp. isolate bog_230	46
Granulicella sp. isolate bog_369	20
Granulicella sp. isolate bog_405	60
Granulicella sp. isolate bog_445	49
Granulicella sp. isolate bog_115	29
Granulicella sp. isolate bog_149	46
Granulicella sp. isolate bog_95	25
Granulicella sp. SCN 62-9	24
Granulicella sp. isolate bog_395	63
Granulicella sp. S156	3
